# Cultural adaptation considerations of a comprehensive housing outreach program for Indigenous youth exiting homelessness

**DOI:** 10.1177/13634615221135438

**Published:** 2022-12-25

**Authors:** Jessie I. Lund, Elaine Toombs, Christopher J. Mushquash, Victoria Pitura, Kaitlyn Toneguzzi, Tina Bobinski, Scott Leon, Nina Vitopoulos, Tyler Frederick, Sean A. Kidd

**Affiliations:** 1 Department of Psychology, 7890 Lakehead University, Canada; 2 Dilico Anishinabek Family Care, Fort William First Nation, Canada; 3 Northern Ontario School of Medicine (NOSM), 7890 Lakehead University, Canada; 4 Thunder Bay Regional Health Research Institute, Canada; 5 Wellesley Institute, Canada; 6 7978 Centre for Addiction and Mental Health, Canada; 7 Department of Criminology and Justice, 85458 Ontario Tech University, Canada; 8 Department of Psychiatry, University of Toronto, Canada

**Keywords:** Canada, cultural adaptation, homelessness, indigenous mental health, organizational cultural competence, youth

## Abstract

Generalist health interventions that aim to reduce chronic health disparities between Indigenous and non-Indigenous populations can be culturally adapted to better meet the needs of Indigenous people in Canada; however, little is known regarding best practices in implementing these adaptations. The present study first provides a review of the research process used to adapt a previous evidence-based housing initiative for Indigenous youth in Northwestern Ontario. Second, it includes an overview of the adaptations that were made and the associated rationale for such adaptations. Third, it examines the experiences of participants and staff involved in the cultural adaptation of the Housing Outreach Program Collaborative (HOP-C), a health intervention re-designed to improve physical and mental health outcomes, wellbeing, and social support for formerly homeless Indigenous youth as they secure housing. Qualitative feedback from interviews with 15 participants and eight program staff, in addition to one focus group with an additional six frontline workers, described perceived outcomes of the program's cultural adaptations. Modifications to the overall program structure, specific roles within the program (including counseling services, peer mentorship, cultural services, and case management), and adaptations to general implementation within the health organization providing the intervention were described by participants and staff as effective and helpful adaptations. The focus of Indigenous values at an organizational level led to consistent adaptations in counseling and case management to best meet the unique needs of the youth involved. Based upon participant interviews, recommendations to future adaptations are provided.

The majority of evidence-based health interventions are not developed specifically to address Indigenous health needs and do not typically incorporate Indigenous views of wellness, including holistic views that address emotional, spiritual, mental, and physical notions of wellbeing (Assembly of First Nations & Health Canada, 2015). Given that there are more health interventions designed for non-Indigenous populations in Canada than for Indigenous populations, one way to facilitate effective outcomes for Indigenous communities is to adapt existing programs to specific population needs. These adaptations utilize effective Western programs and interventions that are evidence-based and are broadly aligned with Indigenous values and goals, while simultaneously considering the unique needs of Indigenous peoples. Such practices exemplify the importance of embedding Indigenous culture throughout interventions, rather than solely adding it in adjunct to Western programming. These adaptations reflect the concept of Two-Eyed Seeing, which is founded on the notion that Indigenous and Western knowledge can be simultaneously considered, valued, and utilized to best address problems (Iwama et al., 2009). Rather than “re-inventing the wheel,” culturally based program adaptations can meet the needs of communities in a way that prioritizes resources and applies best practices previously found to be efficacious within other settings.

Cultural adaptations within health care programming for Indigenous peoples have demonstrated some success over recent years. For example, a Canada-wide program, *ACCESS Open Minds*, that aims to address youth mental health needs, has been successfully adapted to support youth living in the First Nation community of Eskasoni, Nova Scotia, with promising results (Hutt-MacLeod et al., 2019; Mushquash, 2019). Similarly, *The Fourth R*, an evidence-based program designed to promote healthy relationship skills, positive mental health, and prevent violence, was adapted to specifically support the needs of Indigenous youth through a focus on cultural identity development, mentoring, and cultural teachings from within the community (Crooks et al., 2018). This intervention has seen significant gains in mental health outcomes and cultural connectedness in the youth involved (Crooks et al., 2017). A prominent integrative substance use and trauma intervention, *Seeking Safety*, was adapted to support treatment with Indigenous peoples by approaching adaptation through the use of Two-Eyed Seeing, consultation with Elders and an Aboriginal advisory group, as well as the incorporation of traditional healing practices (Marsh et al., 2016). Individuals who received the cultural adaptation of *Seeking Safety* reported that the incorporation of traditional Indigenous healing methods, the presence of Elders, and the sweat lodges were all beneficial to their treatment. These examples demonstrate the feasibility of adapting previously non-Indigenous health-based programs for Indigenous populations, and the diversity of methods used to integrate Indigenous perspectives of health with a Two-Eyed Seeing approach.

Although some programs have adapted previous content to be more suited to Indigenous needs, few document how cultural adaptations are specifically created and implemented. Further, when cultural adaptation does occur, few published trials have assessed the utility of such changes for Indigenous populations. Without rigorous evaluation through various Indigenous knowledge systems, it is possible that, although a program is modified, these changes do not improve health outcomes for Indigenous populations. Therefore, documentation of methods used to adapt programs can not only provide insight into what methods are or are not helpful but can also be used to increase the success of cultural adaptations of future health programs for Indigenous populations.

There are several approaches to cultural adaptations documented in the literature. These approaches include a focus on how to culturally adapt the content of an intervention, as well as discussions on the overall process of adaptation (Baumann et al., 2015). McIlduff et al. (2020) provide an overview of models that delineate how to effectively implement a cultural adaptation. Notably, the Model of Native Healing (Johnson, 2006) provided a model for adapting programs to be culturally integrated for Native Americans. This model emphasizes an approach to the adaptation of Western evidence-based interventions that integrate Indigenous values and traditions (e.g., making the medicine wheel the foundation of the healing model, allowing for a focus on traditional values and healing to create balance and harmony), including an integration of both the cultural and sociohistorical context experienced by the communities it is designed to serve (Johnson, 2006).

More recently, McIlduff et al. (2020) developed the Model of Engaging Communities Collaboratively (MECC) to inform how best to implement evidence-based interventions with Indigenous populations. The MECC includes the following: a thorough consideration of cultural context; a research collaboration founded on mutual respect, trust, and empowerment; adaptation and implementation with community collaboration; adaptation centered around community-identified concerns and solutions; collaborative community consultation; engaging community members/leaders/organizations for additional input; identification of cultural traditions, values, and beliefs; considering ecological fit and sustainability; and collaborative and ethical dissemination of findings.

## The Housing Outreach Program Collaborative (HOP-C)

The Housing Outreach Program Collaborative (HOP-C) is an example of a health intervention that was designed predominately for urban non-Indigenous youth but has since been adapted to better meet Indigenous youth needs. HOP-C is a tertiary preventative intervention that offers case management, individual and group mental health interventions, and peer support to youth exiting homelessness, with a focus on preventing the reoccurrence of homelessness (Kidd, Thistle, et al., 2019; Vitopoulos et al., 2017). HOP-C has seen gains in the involved youth, including improvements in housing stability, mental health, employment, and education (Kidd, Vitopoulos, et al., 2019).

There is a gap in the research literature surrounding evidence-based programs that have been culturally adapted to support the unique needs of Indigenous youth experiencing homelessness. Indigenous peoples disproportionately experience both poverty and homelessness and Indigenous youth are at particularly high risk (Patrick, 2014). Among a national sample of youth experiencing homelessness in 2019, 32% were Indigenous (Gaetz et al., 2019) despite Indigenous youth aged 15–24 representing approximately only 6% of youth in Canada (Statistics Canada, 2017). The outcomes of colonial practices enacted on generations of Indigenous peoples appear to be linked to the high rates of Indigenous youth homelessness seen in Canada. For example, in 2019, 44% of Indigenous youth experiencing homelessness felt they were not connected to their Indigenous culture or community and 69% did not feel connected to a specific area of Indigenous land (Gaetz et al., 2019). Further, Indigenous youth experiencing homelessness are more likely to develop a mental health problem or addiction than non-Indigenous youth experiencing homelessness (Kidd et al., 2017). They are also more likely to have experienced physical assaults, overdose requiring hospitalization, and suicide attempts while homeless compared to non-Indigenous youth (Kidd, Vitopoulos, et al., 2019).

To address this need, the HOP-C program was adapted and implemented by an Indigenous organization servicing 13 First Nations communities in Northwestern Ontario, in partnership with a collaborative research team including the creators of the original HOP-C program. This adaptation was designed to support Indigenous youth experiencing homelessness in Northwestern Ontario (titled HOP-C North). HOP-C North was a six-month intervention that was delivered during a critical time period as youth were transitioning out of homelessness and into stable housing. Much like the original HOP-C program, HOP-C North aimed to address two major components seen as necessary for youth who have recently received housing: (1) general support and information and (2) mental health. These two components were provided through the use of four types of support: transitional case management, group and individual mental health services, peer support, and with the addition of a cultural wellness mentor. The program demonstrated positive outcomes, including increased educational enrollment, attainment of employment, reduced hospitalizations, and increased engagement in clinical mental health services (Toombs et al., 2020).

With the continued pursuit to best support Indigenous youth and adults with mental health, addiction, and social determinants of health needs, documentation of the process by which adaptation occurs is needed. Each Indigenous community has unique needs and thus implementation of culturally based interventions must take place collaboratively to determine relevant needs for the targeted participants in the service. A consideration of the methods needed to carry out such collaborations as well as the means to continuously modify and follow up with the function of the program is also needed. Indigenous youth homelessness is an avenue to consider these methods, as novel interventions that best support these marginalized youth so they can exit homelessness successfully are of primary importance.

## Study purpose

The HOP-C North program generated positive outcomes for Indigenous youth (Toombs et al., 2020), however preliminary analysis did not confirm whether the outcomes were related to any cultural adaptations made to program content and structure. It was hypothesized that the content modifications increased engagement with youth, however a secondary analysis of these modifications is required to fully understand their effects. As such, the purpose of this study is threefold. First, we provide a review of the research process used to adapt a previously evidence-based housing initiative, the HOP-C program, for Indigenous youth living in Northwestern Ontario. Second, we provide an overview of the adaptations that were made, along with the associated rationale for such adaptations. Third, we examine qualitative feedback from participants and staff related to their experiences, and the reported perceived outcomes of these adaptations. These findings are used to inform recommendations for future comprehensive housing outreach programs and related cultural adaptations for Indigenous youth.

## Method

### Research paradigm

The HOPC-North program was originally evaluated for effectiveness in adequately addressing the needs of Indigenous youth and its ability to support them in maintenance of newly secured housing (Toombs et al., 2020). This evaluation of the project has since provided the opportunity to ensure the program adaptation occurred successfully, leading to the purpose of the present study. The implementation of the HOPC-North program was guided by a four-tiered partnership between local First Nations communities, the board of directors of the Indigenous-led organization implementing HOPC-North, a research advisory within the organization, and a research team with a longstanding relationship with the Indigenous organization, as well as a team that included the creators of the HOP-C program. The board of directors represents community needs and involves many band councilors and chiefs from partnering communities. The research advisory communicated with the research team regularly, who would update the board of directors regarding the status of the research and would communicate back to the research team regarding any feedback or changes to be made. The research team also sought ongoing consultation from the Youth Advisory regarding the study methods. The communication across levels of partnerships assured that the research done to evaluate the program was consistent with community values. The consultation process also aimed to ensure that the First Nations communities involved had control over the methods used, consistent with the Ownership, Control, Access, and Possession (OCAP^TM^) standards set by the First Nations Information Governance Centre (2014). The research methods also followed Wilson’s (2008, p. 77) Indigenous research framework, which is based on Indigenous research values (e.g., the responsibility to ensure respectful and reciprocal relations) suggested by Weber-Pillwax (2001). Ultimately, the research methods were approved by both the Indigenous organization research advisory and the Lakehead University Research Ethics Board.

### Participants

#### Program participants.

There were 15 research participants in the HOP-C North program and the ages ranged from 16 to 24 (*M* = 18.80, *SD* = 2.5). Eleven participants (73%) were female, nine (60%) identified as Indigenous, while six (40%) identified as mixed heritage or other. Eleven (73%) identified as heterosexual, while four (27%) identified their sexual orientation as “other.” Among participants, age of first becoming homeless ranged from six to 18 (*M* = 14, *SD* = 3.44) and the mean number of times experiencing homelessness was 4.5 (*SD* = 2.63). Among the participants, 15 engaged in initial interviews at the beginning of the study. Eight participants completed follow-up interviews after a six-month time interval. The participants who did not complete the follow-up interviews had remained in the HOP-C North program but chose not to participate in follow-up research.

#### Staff participants.

Within the HOPC-North program, there were two managers, one peer mentor, two cultural wellness mentors, two youth coordinators (formally called case managers in the original HOP-C model), one clinical social worker, and six front line workers.

### Qualitative outcome interviews

#### Participant interviews.

Fifteen participants completed initial interviews, of which eight completed second follow-up interviews approximately six months after their initial interview.

In the initial interviews, participants provided demographic information and prior difficulties with housing. They were also asked questions regarding their goals for the HOP-C North program, and what they looked forward to about the services provided. The second interview included questions regarding relevant outcomes to the HOP-C North program and questions related to the services the participants used through the HOP-C North program. This included questions about the aspects of HOP-C North participants engaged with, including case management, peer mentorship, group therapy and skill-building sessions, individual therapy, and cultural mentorship. Participants were asked what supports they found helpful, what supports they did not find helpful, and feelings toward their participation in HOP-C North as a whole.

#### Staff interviews.

Eight individual interviews with staff were conducted. Specifically, two managers, a peer mentor, two cultural mentors, two youth coordinators, and one clinical social worker all participated in individual interviews. Among staff who reported their ethnicity, 50% self-identified as Indigenous. Individual interviews involved questions about their role in the HOP-C North program, specific challenges they encountered in trying to fulfill their role, the effectiveness of the program as a whole, and what factors of the program they felt influenced participant outcomes. A focus group was also conducted with six front line staff. Staff participants were asked to reflect on the youth outcomes associated with the program, including the status of their housing, employment, education, mental health, and applied life skills for the duration of the program, and what aspects of HOP-C North may have contributed to these outcomes.

### Thematic analyses

Thematic analysis was completed with aggregated data compiled from both HOP-C North initial and follow-up participant interviews as well as staff interviews completed following six months of program implementation. The interviews and focus group were transcribed orthographically, to preserve naturally occurring speech patterns of participants. Open coding procedures congruent with Braun and Clark's (2006) six-step method for thematic analysis were used: 1) data familiarization, 2) generating initial codes, 3) searching for themes, 4) reviewing themes, 5) defining and naming themes, and 6) describing results.

Two coders initially reviewed the transcripts independently and generated initial codes through open-coding procedures. While open-coding procedures were used during the initial phases of data analysis, the HOP-C North framework components were referenced to pull out themes as they related to case management, mental health support, peer mentorship, and cultural mentorship, as these aspects were key programming components of HOP-C. The use of a deductive approach allowed data to be generated that described both youth and staff experiences related to these program components, and aligned with the overall goal of the study.

Identification of researcher reflexivity and contextual influences are listed as two standards reporting within qualitative research designs (O’Brien et al., 2014), and such practices are particularly relevant when engaging in research processes with Indigenous communities (Toombs et al., 2019). Given that initial data synthesis and analysis was completed by two non-Indigenous reviewers, this step was essential to ensure that results accurately reflected community, contextual, and cultural values and representation. These standards were addressed in two ways. Firstly, after each coder had identified initial themes from the transcripts, these themes were compared and discussed among the researchers, to ensure inter-rater reliability and accuracy. Secondly, these themes were collaboratively reviewed with the Indigenous organization leadership and staff. The utility, relevance, and accuracy of the themes were determined through consensus of members of the Indigenous-led Research Advisory Board, and no themes proposed to the Advisory were refused at this stage.

## Results

### Preliminary program content adaptation

The adaptation of the HOP-C program began through an ongoing research collaboration with the creators and original implementers of the program in a larger city center in Ontario and the Indigenous organization aiming to implement the adapted program. The original program was chosen as one that had an epistemological framework that was already perceived to be well suited for Indigenous youth. For example, the flexibility in programming and the housing first modality contrasted other interventions that could have been adapted for Indigenous youth. Moreover, the program had been developed and already evaluated among a diverse urban population in the Greater Toronto Area (Vitopoulos et al., 2018). Content modifications were discussed and decided upon among the research advisory of the Indigenous organization, with expert-level content reviewed by those well versed in the needs of Indigenous youth in the surrounding areas. This included those with extensive experience in mental health and addiction services, child welfare, and housing support services with Indigenous youth in the community. The research advisory also includes team members who have a strong understanding of the history of Indigenous peoples, including how colonialist practices and subsequent loss of culture are intrinsically linked to the high rates of Indigenous youth homelessness, as well as the barriers these youth face in securing and maintaining housing and accessing services.

Program adaptation considerations involved 1) how to best incorporate cultural teachings and Indigenous notions of wellbeing and healing 2) and how to implement the program in a different urban setting, with considerably different resources and needs. This led to specific changes related to both the overall style and structure of the program, but also modifications to the specific roles of staff involved. These changes were theorized to create positions that best supported Indigenous youth needs in the community. These are described in Table 1.

**Table 1.** HOP-C North program adaptations and associated rationale.

Program Content	Adaptation	Rationale
Overall Structure and Delivery Style	Implementation occurred within one organization rather than a community-wide collaboration.	HOP-C North was limited to one organization due to the expertise and availability in the community.
	Youth involved in the program were identified based on prior agency involvement and need.	Indigenous youth involved in the child welfare system are at increased risk for homelessness, and thus the host organization was able to support those identified as in high need.
	Added additional core service of cultural wellness mentor.	A cultural wellness mentor would ensure that youth had access to cultural support (e.g., facilitating smudging, attending a sweat lodge or sharing circle) whenever needed, and that other providers would be able to maintain that their work was grounded in Indigenous knowledge.
	Prioritized hiring Indigenous staff and/or those with knowledge of Indigenous culture.	To ensure Indigenous culture (e.g., approaches to mental wellness that include a balance of the mental, physical, spiritual, and emotional and prioritize purpose, hope, belonging, and meaning) was embedded in all facets of HOP-C North programming.
Role of Case Managers	Change in name to “youth coordinator.”	Change in language to support the philosophy that the youth involved were autonomous in their decision-making.
	Increased emphasis on relationship-building before case management.	Many Indigenous youth have had complex relationships with service providers (i.e., through the child welfare system), and thus significant trust-building was needed.
	Maintained level of communication regardless of case management needs.	Case managers would check in with youth involved, regardless of the need for case management, to provide youth with a consistent and trustworthy relationship.
	Case managers with knowledge of Indigenous teachings and practices.	Case managers’ knowledge of Indigenous culture allowed them to incorporate teachings and practices on a case-by-case basis (e.g., referencing Anishinabek teachings to support youth with a given problem; connecting with Elders to facilitate youth in receiving their Spirit names).
Role of Counseling Staff	Counselors attended all groups (including non-mental health related).	The counselor's attendance at all skills groups was done to increase familiarity and comfort with the counselor among the youth.
	Youth had access to counselors on an as-needed basis.	An open-door policy to counseling was viewed to decrease barriers for the youth.
	Flexibility with location of therapy.	The counselor's flexibility in locations used was done to avoid any location-specific barriers.
	Incorporation of Indigenous teachings into interventions provided.	The counselor incorporated Indigenous teachings into interventions (e.g., using the medicine wheel when discussing coping strategies) used to support youths’ cultural identity in relation to their wellbeing.
Role of Peer Mentors	Peer mentors were not hired from outside of the program.	Best practices for incorporating Indigenous peer mentorship positions are not clear; the host organization wanted to approach peer mentor involvement carefully.
	When youth involved in the program felt ready, they were hired into peer mentor positions.	Youth who progressed through the program were moved into peer mentorship positions for two reasons: they understood the program and had lived experience; it provided the peer mentors a chance to develop in a leadership position.
	Flexibility in what each individual peer mentor's position looked like.	Staff worked with mentors to tailor job requirements that were feasible for each youth and their unique needs.
Role of Cultural Wellness Mentor	New position added to HOP-C North.	This role was added as cultural wellness was viewed as a necessary support to provide to youth.
	Worked collaboratively with other supports and with organization's cultural team.	Prioritization of collaboration with the other supports to ensure that Indigenous culture was embedded in all facets of the HOP-C North program.

### Process of agency adaptation

The cultural adaptation of the HOP-C program occurred through three levels of implementation: organizational, staff, and individual (see Table 2 for a brief summary of each level of implementation). Cultural adaptation that occurred at an organizational level included that the organization implementing HOP-C North was Indigenous-led, self-governed, and guided by Indigenous values and traditions. At the staff level of implementation, staff were selected based on their understanding of Indigenous youth needs and their ability to provide the various components of the HOP-C program (case management, mental health, peer mentorship, cultural wellness mentorship) through a cultural lens. At the individual level, cultural adaptations were made for the clients engaged in the program. This included modifications to service provision to make engagement culturally safe for the youth and that supported their cultural identity, autonomy, and experiences at every point of engagement.

**Table 2.** Specific strategies of program adaptation at each level of implementation.

Implementation Level	Program Modification
1. Organizational	Respectful collaboration between partnering organizations Inclusion of standardized training Embedding culture into all activities
2. Staff	Focus on hiring Indigenous staff who are connected with culture and felt comfortable teaching skills Staff were hired who had experience working with Indigenous youth in the community
3. Individual Participant	Creation of youth advisory board Seeking program feedback in all services, formally and informally Implementation of program content modifications

#### Organizational level.

Organizational-level adaptation aimed to ensure that an Indigenous-led organization saw the HOP-C program as a means to meet the needs of their partnering communities. Beyond this, the organization already provided ongoing standardized training to all staff related to Indigenous experiences of health and wellbeing. This includes a comprehensive education of the history of Indigenous peoples, the effects of colonization, and the ongoing trauma, marginalization, and poor socio-economic conditions faced by many Indigenous communities. The organization also utilizes the First Nations Mental Wellness Continuum Framework (FNMWCF; Assembly of First Nations & Health Canada, 2015) as a framework to guide all service provision. This includes priority towards supporting hope, belonging, meaning, and purpose among Indigenous peoples. The organization values engagement in cultural activities and knowledge-sharing, including organization-wide opportunities for smudges, sweats, pow-wows, and land-based activities.

#### Staff level.

After implementation was met through support at the organization level, staff were selected to be part of the HOP-C North team who were able to carry out HOP-C programming using culturally relevant methods. This included providing staff with the education needed to understand the unique needs of Indigenous youth experiencing homelessness, and the unique barriers they face related to service engagement (e.g., past experiences of racism, lack of continuity of care upon aging out of foster care). Many staff identified as Indigenous and were engaged in cultural and spiritual activities regularly, and thus were able to share their knowledge regarding these activities. In this way, staff simultaneously served as leaders in demonstrating how they utilize cultural activities as a means to support their own wellbeing. Staff who had experience working with Indigenous youth in the community were also hired, including youth who had been involved in the child welfare system and/or had experienced homelessness.

#### Individual participant level.

Individual-level adaptations included program-specific content adaptation (as outlined in Table 1), as well as novel methods to monitor participants’ experiences in the program in order to make changes as necessary. The agency created a youth advisory board to help guide activities. Typically, the youth advisory would guide the project from start to finish, but in this program, it was adapted to be developed simultaneously as the program was rolled out. Participants who were showing success and meeting their individual goals were invited to be part of the board so as not to overwhelm them.

### Participant outcomes of cultural adaptation

Themes that emerged from qualitative interviews from both participants and staff are described below (see Table 3 for summary). These data relating program modifications to youth experiences of the program were generated from a secondary analysis of general program outcome data (Toombs et al., 2020). Specific youth and staff experiences or preferences relating to each program modification were not explicitly asked during interviews. Rather, these were generated from analysis of more general qualitative data, where experiences in the program more broadly were evaluated. As a result, some program modifications, although with sound rationale for inclusion, were not explicitly mentioned by staff or youth participants and are thus not associated with specific program outcomes.

**Table 3.** Reported outcomes from program content modifications from staff and youth participants.

Program Content	Adaptation	Reported Outcome by Youth/Staff	Representative Staff Quotations	Representative Participant Quotations
Overall Structure and Delivery Style	Focus on hiring Indigenous staff	Increased understanding of cultural needs embedded in service delivery	“The way in which we deliver services is very much rooted in the values and traditions and customs of Indigenous people to the point where the titles of the team members and the roles of the team members were adapted to suit the cultural needs of our youth up here.” “It was beneficial for the program to be delivered by an Indigenous organization who really understands the historical experiences of Indigenous people.”	“They’re really nice, I enjoy them. We do sage cleansings, and I have cedar from them. We do traditional teachings, and meal preparations. It's nice to attend.”
	Flexible service delivery (including providing rides, changing locations of services, and offering multiple groups per week)	Access to support as needed Allowed youth to seek services they would like to access Allowed youth to develop activities they wished to partake in	“You need to strike while the iron is hot. And like you said, a lot of them lose that motivation as time goes on, or they find alternative coping skills that maybe aren't the healthiest. So, if you can get in there right away and break down that barrier of the waitlist, it's super beneficial.” “I think offering that [services] is super important, but then I think making sure that we balance not pushing it on people, because we sometimes lose people for that too.”	“She would always help me with things, like small stuff. Say hey, [respondent], what do you need help with, this, or need that. She would come help me cook, and she would help me, guide me on what to do. She's like a teacher, almost.” “It's just a really comforting … I don't feel … I don’t know, I don’t feel targeted at all. I don’t feel pressured to be there. Like I said, it's very welcoming, so you can relax. It's good.” “Not everyone wants to go to do a program. They just want to be settled in a house, or they want to be settled in a program where they know if they need help they’ll call for it, but they just don’t want to tend to a bunch of stuff.”
	Ongoing collaboration within specific roles of team members	Better services for youth through ongoing staff communication Avoiding duplication of services	“I think all of the components are essential. Again, you need a well-functioning team. I don’t think it's necessary for improvement, but I just think it's necessary for that coordination aspect of the team is really, really essential to the success of the program overall. Because there's so much flexibility for youth to kind of participate in different components and the youths’ needs change quite a lot over time.”	“Well, the staff are there to listen if you really need to or they could provide you with support in any kinds of ways you need” “Just having regular real conversations with them.”
Role of Case Managers and Counseling Staff	Staff participation in all youth activities	Increase of staff familiarity with youth to build rapport More casual access to services for youth	“These youth have to come to you for an appointment, but you meet them where they’re at, which is very different from what lots of people are used to. They’re used to more of an office setting where the youth will come and meet with them because your appointment is at 2:00, but with the HOP-C piece, what we really pushed was the worker went to meet the youth where they were at. So, that was meeting them at school, meeting them just at our [housing] space, whatever, so really worked a lot on that.”	
	Staff with previous relationships with youth	Relationship-building and rapport was easier to build and maintain Authenticity in relationships Staff understanding of youth needs.	“Initially, a few youth had connected with another staff and they really clicked very well because they might have worked together in the past or their personalities really meshed. And then when you have a new worker coming in, it's kind of hard to rebuild that relationship and that trust, right, because now it's like, oh, there's another worker, there's another worker, are they really here for me?” “When we did have new staff come in, it was really good to have her connected with the new staff so then she could be like, heads up, this is what works for this young person, this is what doesn't work, maybe you have to change your approach as to how you work with this person. So, that really made a difference.”	“Oh, like I said before, I knew [HOP-C worker] since, like, 2010. She's been working with [Agency Name] for a long time, so. That's how I was able to communicate with her. [HOP-C youth worker], I met her a couple of years ago, like in 2015, 2016, because I always used to attend those [Agency Name] meetings.” “But that's [HOP-C worker]. It doesn't even feel like case management. It just feels like [HOP-C worker] helping me because she has known me for eight or more years.”
	Providing services in youth living space	Immediate access to services	“We’re basically in their house, they come downstairs and we’re there.” “… we had staff visible in that space that they could connect with when they needed.”	“More of a support because she was there too with us, she was like a mom at the house.”
Role of Peer Mentor	Embedded process of selection (ongoing or program participants were chosen)	None reported	None reported	None reported
Role of Cultural Wellness Mentor	Creation of this position	Opportunity for staff and youth to learn more culture, connect with Elders, and engage in cultural teachings as desired Regular access to cultural mentorship	“I think the culture is a very individual basis. I think that we offer it, just in general, is relevant … to me, culture isn't about beads and feathers, here, let's make a dream catcher, it's about how you do your practice. So, honesty, respect, and teaching about the seven gifts in more of how you do your work.” “Throughout the city, you have to have multiple referrals, and also for you to connect with an elder takes quite a while, even if you go to different agencies, and for them to make that link. And I think having that direct staff available for the youth made it that much easier for them to reconnect with culture.”	“They’re respectful, they don’t make me feel stupid for not knowing something. They’re supportive.” “I really like the teachings. I like the welcoming feeling that comes along with group. I just enjoy being a part of that community now. I feel like they’re doing things really well. Because when I first started, like I said, I did not want to be a part of it whatsoever, I was completely miserable. But I do enjoy it a lot now, so I feel like they’re definitely doing well.” “Oh, I seen [HOP-C cultural mentor] last week, she's still … I still talk to her. I’m like, hey, [HOP-C cultural mentor], how have you been. She's no stranger to me. If I see her, I’m definitely going to greet myself to her.”

#### Program structure.

The prioritization of Indigenous values was mentioned as an important factor that helped to increase the acceptability of the HOP-C North Program, particularly given that the program was implemented within an Indigenous health organization, by many Indigenous staff. Within the program, youth were given the opportunity to step into leadership roles as a means to support their autonomy and self-determination. Staff, who often had longstanding professional relationships with program participants, were flexible with service provision. This included providing rides to programming when needed, where public transportation was a difficulty for youth often due to past experiences of discrimination. Counseling was often offered in spaces where the clients felt comfortable (e.g., in their building), recognizing that offering services in certain areas of the community would serve as a significant barrier (due to prior negative experiences in that area of the community). One staff member noted that “I think being in their living space made a huge difference.”

The availability of staff also reduced barriers as participants felt they could access support when needed. This was cited to make the program a good fit with Indigenous youth who often had many experiences of inconsistent service providers and caregivers in the past. One staff member noted:

we had the essential components of the program and we delivered those, but there was a lot of capability to adapt the program to meet the needs of the local population and I think that that is essential anywhere you take a model and you deliver it in a different area.

This is further exemplified by one participant who explained: “Yeah, it wasn't like, oh, I see my counselor once a week, I go to her office and I sit there. I was actually involved in helping out with programming and just having regular real conversations with them.”

Overall recommendations around program structure by staff and program participants were related to the continued delivery of flexible content in central areas of the city, including locations that are more central to bus stops and areas of town that were perceived to be safer by participants. At the beginning of the program, staff role responsibilities were not completely defined, which prompted staff to suggest the issue should be addressed prior to future implementation. Given that relationship-building was mentioned by staff as an important step toward engaging youth within the HOP-C North programming, some staff suggested that staff turnover within the organization was a challenge to program implementation.

#### Clinical counselors and case managers.

Staff understood that the youth needed nurturance, flexibility, and patience as they navigated out of homelessness. As such, programming, case management, and counseling were all provided in a flexible manner to meet each youth's individual needs. This was identified by one staff member who expressed that “our participants seem to have higher mental health needs or more significant mental health needs than the participants in the South so we were dealing a lot more with intense crisis and so that disrupted participation.” There was no expectation regarding the level of engagement youth would need to have with the program in order to be involved. It was understood that it was therapeutic for the youth to know they had access to case management and mental health support on an as-needed basis, rather than demanding a certain level of consistent engagement in order to access the services when truly needed. This was viewed as important to building relationships with youth, many of whom had had inconsistent relationships with service providers in the past. For example, one participant noted “But that's [HOP-C worker]. It doesn't even feel like case management. It just feels like [HOP-C worker] helping me because she has known me for eight or more years.”

Some challenges reported by staff and youth participants were related to understanding more of the group dynamics of the program, including having a set process to determine how prior relationships may affect group dynamics. Some program participants were known to each other, which affected their interest in group participation at times. One participant relayed that when groups were closed, rather than open, participants were able to build and maintain supportive peer relationships in that group, and that having different group content delivery styles was helpful to engaging youth. When clinicians were meeting youth at places they requested, one staff voiced, “I think it's been a challenge to find little places throughout the city where there is confidentiality that clients are willing to come to.”

#### Peer mentor.

Most participants did not utilize services within the peer mentorship program components, and therefore, specific outcomes were not generated. As many staff hired in additional roles were Indigenous people, some with lived experiences of adversity, it is possible that some benefits typically facilitated through engagement in peer mentorship were shared across other staff roles. As one staff member stated:

I’m third generation residential school and I’ve been through a lot myself personally and so I can identify with these youth, what they’re going through or have gone through, and so can some of my other co-workers. So, it's just understanding because I can say to them, I’ve been where you are, I’ve lived it and I overcame it, you can too.

When asked about how to improve the peer mentorship program, staff relayed that having a greater diversity of team members could improve the connections that current program participants formed within this role. As stated by one participant, “to get everybody to come together all the time and to be able to have that one peer that they really would connect with, that was a struggle.” Having a greater number of peer mentors could also help reduce potential burden on this position to fill the role and share responsibilities with more mentors. One staff member noted that offering greater financial incentives for peer mentors may encourage participation.

#### Cultural wellness mentor.

In addition to the original HOP-C program areas (case management, mental health, and peer support), a cultural mentor was also included in the HOPC-North staff team in order to support both youth involved in the program as well as staff on an as-needed basis. The cultural mentor also worked with staff employed in mental health and case management to support the integration of cultural teachings and activities into these services. Staff were able to connect youth to an Elder (female or male) if that was something youth were interested in. Staff valued the way in which culture was embedded in their service delivery, as noted by one staff member: “it seemed like having that integration piece, with having culture there, really balanced everything out.” Many youth had never been exposed to Indigenous culture before. As such, the opportunity to learn about their culture was provided, though youth chose their level of engagement. This was fluid, such that over time, many youth engaged more in cultural activities as they felt comfortable doing so. They were provided opportunities to explore their cultural identity and received their spirit names. One participant noted:

I really like the teachings. I like the welcoming feeling that comes along with group. I just enjoy being a part of that community now. I feel like they’re doing things really well. Because when I first started, like I said, I did not want to be a part of it whatsoever, I was completely miserable. But I do enjoy it a lot now, so I feel like they’re definitely doing well.

## Discussion

The goals of the present study were to describe the methods used to adapt an evidence-based comprehensive housing support program to meet the needs of Indigenous youth, capture the specific adaptations that were made, and provide a qualitative analysis of staff and participant perspectives on the utility of these changes in adequately supporting the unique needs of the youth. While some programs have been adapted to better address the needs of Indigenous peoples, there is little documentation on the processes involved to ensure successful adaptation. A consideration of methodology and analysis of the successful components of adaptation can help support future programs as they attempt to adapt existing programs to best support Indigenous peoples.

There were many specific processes that took place to adapt the HOP-C program to better support the needs of Indigenous youth exiting homelessness. First and foremost, the HOP-C program was selected as a viable program due to its original program structure, such that prior to adaptation, it was already designed with flexibility to meet the needs of marginalized populations. After HOP-C had been chosen as the program that would be adapted, there were specific methods used in order to decide upon appropriate adaptations. The success of a new intervention within a health care agency is related to how a program is suited to the agency's needs, but also how it is implemented within that agency. Although cultural adaptation often involves the modification of program content, it also involves modification of organizational structures in order to adequately implement a new program. Adapting processes within an organization to reflect cultural adaptation, even one that is Indigenous-led, can affect the success of an intervention. Ultimately, specific strategies were implemented to achieve successful adaptation at the organizational, staff, and youth level.

While the current adaptation was conducted prior to the publication of the MECC (McIlduff et al., 2020), the adaptation processes used in this study were congruent with many of the core aspects of the MECC. This was due to the collaborative research partnerships between the research team, the Indigenous community partner, and their research advisory that were all grounded in OCAP principles. Indeed, the adaptation involved significant attention paid to the cultural context of the youth it was being adapted for, as well as the cultural traditions, values, and beliefs of the Anishinabek people. The adaptation also involved partnerships founded on respect and trust, as well as ongoing collaboration and consultation with the surrounding communities’ leadership regarding the project aims and meeting the needs of their communities. Dissemination of findings followed OCAP principles and was at the discretion of the Indigenous partner. Notably, the MECC also includes consideration for ecological fit and sustainability of an adapted intervention, which was a challenge of the current adaptation due to funding constraints.

Changes to the original HOP-C program were implemented in the overall structure of the program, as well as in each area of service provision (case management, mental health, and peer mentorship). Structural changes involved both the prioritization of hiring staff who were Indigenous and/or had a background in Indigenous knowledge and values as well as the inclusion of a cultural wellness mentor as an additional service included in the core support offered in the program. Both these changes supported the notion that Indigenous culture, and supporting the cultural identity of the youth involved in the program, was needed. The role of the cultural wellness mentor was also designated to collaborate with the other staff members in the program to ensure that Indigenous culture would be embedded in each support offered. These changes prioritized cultural wellness as equally important to other support services for youth exiting homelessness, as well as understanding that culture interacted with other services provided (e.g., mental health services could not be offered without understanding and incorporating Indigenous views of wellbeing). Indeed, staff highlighted that making Indigenous culture and values the foundation of the program was ultimately integral to its success.

Content adaptations were also made to case management, counseling, and peer support services with variable success. Changes to case management and counseling prioritized reducing common barriers to care that Indigenous youth experience in the community. Many Indigenous youth who experience homelessness have experienced complex histories with service providers (including child welfare involvement) and trauma (both prior and during homelessness; Kidd, Thistle, et al., 2019), and ultimately may have difficulty developing trusting relationships with service providers. To minimize this, the case managers prioritized relationship-building and flexibility in their approach. Staff viewed this as being a fundamental reason why youth trusted them to support them in helping to navigate services in the community.

Meanwhile, the counselor's role was modified to prioritize patience (through an “open door” policy to therapy, rather than solely offering weekly appointments), flexibility (offering services in locations that were feasible and safe for the youth), development of trust (through the counselor's attendance at many groups), and promotion of cultural identity (through incorporating Indigenous teachings into the interventions used). The youth reported valuing the development of trust, patience, and flexibility of the counselor, though did not explicitly speak to the utility of incorporation of Indigenous teachings into interventions.

Consistent with the original HOP-C model, peer mentorship was also included in the HOP-C North services offered, though was adapted in terms of its implementation. Little is known about best practices for implementing Indigenous youth mentorship as little research has considered this avenue. However, peer mentorship has a longstanding history among First Nations culture, where the mentor–mentee relationship is developed through sharing societal values and cultural practices (Bisanz et al., 2003; Klinck et al., 2005). While the original HOP-C model had hired peer mentors who had successfully sustained housing for over two years and had successful engagement in employment and/or formal education, HOP-C North decided to engage youth who were involved in HOP-C North who then grew into peer mentor roles as they progressed through the program.

The peer mentors at HOP-C North often still faced many difficulties associated with recently securing housing, as well as mental health difficulties and balancing engagement in school and/or employment. The participants in HOP-C North largely did not engage with the peer mentors or note any utility from this program in their interviews. It is possible that the methods used to support the peer mentorship development resulted in this. Ultimately, an understanding of how best to implement Indigenous youth mentorship among those who have experienced homelessness is needed. While the peer mentor position may need refining for future implementation, it is worth noting that peer mentorship positions can serve both to support other youth, but also to support the peer mentors as they develop skills in their respective positions. Staff at HOP-C North noted immense growth among the involved peer mentors, who grew in their confidence and refined their leadership skills.

Notably, the themes that emerged from qualitative feedback regarding the HOP-C North program often did not speak explicitly to the utility of cultural additions (e.g., access to a cultural wellness mentor or engagement in traditional activities), but rather spoke to the overarching Indigenous values embedded throughout the program. This highlights the utility in considering a cultural adaptation from a top-down approach, such that Indigenous value and knowledge systems are understood and implemented from an organizational level all the way down to content-specific adaptations of a program, rather than solely adjunct programming to address cultural needs.

### Recommendations for future cultural adaptations

Through the successes and lessons learned in adapting a multi-component, tertiary intervention aimed at stabilizing pathways out of homelessness among Indigenous youth, recommendations for future adaptations to support Indigenous youth can be gleaned. Such recommendations relate to organizational-level changes, specific modifications to clinical, peer mentorship, and counseling services, and suggestions for future evaluation strategies.

#### Organizational considerations.

Implementing a culturally adapted program involves adaptation at the organizational level. This includes an organizational understanding of Indigenous values, as well as an understanding of the relationship between the history of Indigenous peoples and current rates of Indigenous youth mental health difficulties and addiction, homelessness, and involvement in the child welfare system. Organizations can also consider that research processes should be determined and governed by Indigenous communities, such that the methods for evaluating any implemented adaptations are driven by community needs and are consistent with OCAP principles. This also fosters respectful and reciprocal relationships among communities, organizations, and researchers.

#### Case management and counseling.

Hiring staff with an understanding of Indigenous values and knowledge to hold case management and counseling positions aids in embedding Indigenous culture throughout the program. Moreover, these positions may better support Indigenous youth by prioritizing patience, flexibility, development of trust, and promotion of cultural identity. This may include a fluid job description, as the needs of the youth involved are often unique and may best be supported with novel methods by the case manager or counselor.

#### Peer mentorship.

Careful consideration of how best to implement Indigenous peer mentorship is needed. This includes considering what the goal of the mentor position is, and how such a position can be implemented to support both the targeted youth and the mentor engaged in the position. Flexibility and autonomy in peer mentor positions while simultaneously maintaining role expectations may be better achieved by allowing peer mentors to define their roles in a way that is feasible for them to do while also having expectations that this self-determined role is fulfilled.

#### Adaptation evaluation considerations.

Ongoing evaluation for the utility of the adaptations implemented is required. In the present study, no methods for evaluating specific content adaptations were considered at the onset. As such, it was difficult to assess the effects of specific components of cultural adaptation. Future programs may consider determining means to measure outcomes of each facet of content adapted through pre- and post-analyses. Evaluation can be coordinated through Wilson’s (2008, p. 77) Indigenous research framework, based on Indigenous research values suggested by Weber-Pillwax (2001), as a means to consider how evaluation methods reflect Indigenous knowledge systems and are valuable to the Indigenous communities involved.

Ultimately, in considering these recommendations, it is also worth noting that cultural adaptation is a dynamic process, requiring multiple rounds of adaptations and evaluation of implemented changes. As no health intervention is culturally relevant for every single user, refinement to adapt services for most participants requires ongoing consultation and review.

### Limitations

Despite the strengths of the current study, there are also inherent limitations worth mentioning. First, study attrition was certainly a limitation and may have biased the results, given that almost half of the participants did not complete the follow-up interview. However, study attrition is not uncommon among individuals experiencing homelessness due to the complexity of life difficulties they may be encountering. Second, as this study was a secondary analysis, the qualitative interviews captured many components of the adaptation even though they were not uniquely designed to do so but were rather to capture participants’ overall experience in the intervention, and therefore may not have fully captured participants’ views on the adaptations specifically. Third, many adaptations made to the original HOP-C program could also be viewed as related to strong community-based partnerships and youth-centered work, rather than solely related to cultural adaptations. To that end, successful cultural adaptations for Indigenous youth likely involve collaborative community-based partnerships and youth-centered work to adequately meet the needs of the youth in a culturally informed manner.

## Conclusion

With the continued emergence of culturally adapted health interventions to support Indigenous peoples, an understanding of methods used for successful cultural adaptation, as well as best practices for evaluating the success of cultural adaptation, are needed. In the present study, we examined the methods and outcomes involved in implementing a cultural adaptation of a comprehensive tertiary intervention aimed at supporting Indigenous youth exiting homelessness. Adaptation occurred through organizational processes as well as specific content adaptations. Indigenous values at the core of the program led to consistent adaptations in counseling and case management to best meet the unique needs of the youth involved. More research is needed on best practices for supporting Indigenous peer mentorship. Ultimately, consideration of methods when implementing a culturally adapted program as well as an evaluation of successful components of adaptation can benefit future cultural adaptations to best meet the health needs of Indigenous peoples.
